# Cloning and Functional Characterization of Cycloartenol Synthase from the Red Seaweed *Laurencia dendroidea*

**DOI:** 10.1371/journal.pone.0165954

**Published:** 2016-11-10

**Authors:** Gabriela Calegario, Jacob Pollier, Philipp Arendt, Louisi Souza de Oliveira, Cristiane Thompson, Angélica Ribeiro Soares, Renato Crespo Pereira, Alain Goossens, Fabiano L. Thompson

**Affiliations:** 1 Departament of Marine Biology, Federal Fluminense University (UFF), Niterói, Brazil; 2 Institute of Biology, Federal University of Rio de Janeiro (UFRJ), Rio de Janeiro, Brazil; 3 SAGE-COPPE, Federal University of Rio de Janeiro (UFRJ), Rio de Janeiro, Brazil; 4 Department of Plant Systems Biology, VIB, Gent, Belgium; 5 Department of Plant Biotechnology and Bioinformatics, Ghent University, Gent, Belgium; 6 Grupo de Produtos Naturais de Organismos Aquáticos (GPNOA), Núcleo de Estudos Em Ecologia e Desenvolvimento Sócioambiental de Macaé, Federal University of Rio de Janeiro (UFRJ), Macaé, Brazil; Texas A&M University College Station, UNITED STATES

## Abstract

The red seaweed *Laurencia dendroidea* belongs to the Rhodophyta, a phylum of eukaryotic algae that is widely distributed across the oceans and that constitute an important source of bioactive specialized metabolites. *Laurencia* species have been studied since 1950 and were found to contain a plethora of specialized metabolites, mainly halogenated sesquiterpenes, diterpenes and triterpenes that possess a broad spectrum of pharmacological and ecological activities. The first committed step in the biosynthesis of triterpenes is the cyclization of 2,3-oxidosqualene, an enzymatic reaction carried out by oxidosqualene cyclases (OSCs), giving rise to a broad range of different compounds, such as the sterol precursors cycloartenol and lanosterol, or triterpene precursors such as cucurbitadienol and β-amyrin. Here, we cloned and characterized the first OSC from a red seaweed. The OSC gene was identified through mining of a *L*. *dendroidea* transcriptome dataset and subsequently cloned and heterologously expressed in yeast for functional characterization, which indicated that the corresponding enzyme cyclizes 2,3-oxidosqualene to the sterol precursor cycloartenol. Accordingly, the gene was named *L*. *dendroidea* cycloartenol synthase (*LdCAS*). A phylogenetic analysis using OSCs genes from plants, fungi and algae revealed that LdCAS grouped together with OSCs from other red algae, suggesting that cycloartenol could be the common product of the OSC in red seaweeds. Furthermore, profiling of *L*. *dendroidea* revealed cholesterol as the major sterol accumulating in this species, implicating red seaweeds contain a ‘hybrid’ sterol synthesis pathway in which the phytosterol precursor cycloartenol is converted into the major animal sterol cholesterol.

## Introduction

The genus *Laurencia* belongs to the phylum Rhodophyta (red algae) and is widely distributed across the oceans [1]. *Laurencia* species are found between the intertidal and subtidal zones at a depth of 3m and are recognized as a crucial source of specialized (secondary) metabolites [2–5]. The specialized metabolites that are stored in *Laurencia* species are mainly halogenated compounds that play important ecological roles, such as chemical defence against bacterial colonization and infection [6–9]. Most of the described specialized metabolites isolated from red seaweeds are terpenes, especially triterpenes, sesquiterpenes and diterpenes [10, 11]. Besides ecological roles, these compounds have a wide array of activities and valuable medical properties [9]. Triterpenoids isolated from *Laurencia* species with important pharmacological properties include the squalenoid-derived triterpenoids, thyrsiferol and venustatriol (both isolated from *L*. *viridis*) and laurenmariannol and (21α)-21-hydroxythyrsiferol (isolated from *L*. *mariannensis*) with cytotoxic activity against P-388 cells [12, 13]. Furthermore three squalene-derived brominated triterpenes dehydrothyrsiferol, 10-epidehydrothyrisiferol and isodehydrothyrsiferol isolated from *L*. *viridis* also had cytotoxic activities against cancer cell lines [14].

All terpenes are synthesized from the universal building blocks isopentenyl pyrophosphate (IPP) and dimethylallyl pyrophosphate (DMAPP) that can be generated via either the mevalonate (MVA) or the 2-*C*-methyl-d-erythritol 4-phosphate (MEP) pathway. Most bacteria, including the photosynthetic cyanobacteria, use the MEP pathway to synthesize terpenes. In contrast, animals, fungi, archaea and some Gram-positive bacteria exclusively rely on the MVA pathway for terpene synthesis [15]. It is suggested that plants acquired the MEP pathway through cyanobacterial endosymbiosis and consequently use both pathways for terpene biosynthesis [16]. In plants, the IPP and DMAPP generated through the cytosolic MVA pathway is mostly used for the synthesis of sesquiterpenes and triterpenes, whereas IPP and DMAPP generated through the plastidial MEP pathway are mostly used for the synthesis of all other types of terpenes [17, 18]. Green algae do not possess the genes for the MVA pathway and their sesquiterpene and triterpene building blocks are consequently derived from the MEP pathway [19–21]. However, red algae such as *Laurencia* species retained both IPP pathways during their evolution and use both biosynthetic routes [22].

A key step in the biosynthesis of triterpenes and sesquiterpenes is the sequential condensation of two units of IPP with DMAPP to yield farnesyl pyrophosphate (FPP). For the synthesis of triterpenes, two units of FPP are fused head-to-tail to form squalene, which is subsequently epoxidized to 2,3-oxidosqualene, the direct triterpene precursor [21, 23]. In most organisms, the epoxidation of squalene is carried out by squalene epoxidase [24], however, several species, including the diatom *Phaeodactylum tricornutum*, lack a conventional squalene epoxidase [21].

The great diversity of triterpene structures, with more than 100 different carbon skeletons known in the kingdom of life, is due to different oxidosqualene cyclases (OSCs) and, in particular, to the number of rearrangement steps that the different OSC enzymes can catalyse in the third stage of the cyclization reaction [25–27]. The OSCs belong to a gene superfamily divided into 10 groups according to their product specificity and higher rank phylogeny [28]. They are used both in primary metabolism (e.g. lanosterol/cycloartenol) or specialized metabolism [29, 30].

Some OSC enzymes have more than one final product, e.g. an OSC from *Citrullus colocynthis* produces cucurbitadienol and lanosterol [31]. Furthermore, OSC enzymes can produce precursors for both non-sterol triterpenes, like lupeol, and sterol triterpenes, like cycloartenol [32]. To characterize the enzymatic product, OSC genes are often ectopically expressed in yeast [21, 30, 33–35] or in plants via transient expression in *Nicotiana benthamiana* [36, 37].

To date, approximately 50 OSC genes have been cloned from various plant species, e.g. *Arabidopsis thaliana*, *Artemisia annua*, *Lotus japonicus* and *Oryza sativa* (S1 Table). So far, such studies have not been performed for OSC genes from red seaweeds. In this context, our aim was to clone and functionally characterize an OSC from the red seaweed *L*. *dendroidea* by expression in a sterol-engineered yeast strain.

## Material and Methods

### Identification and Cloning of *LdCAS*

To screen for potential OSCs, we performed a TBLASTX search in the transcriptome assembly of *L*. *dendroidea* [38] using the nucleotide sequence of the characterized *A*. *annua* cycloartenol synthase (*CAS*) (GenBank accession KM670093 [39]) as query. The domain composition of the sequence coding for the OSC from *L*. *dendroidea* was obtained through search for conserved domains using the National Center for Biotechnology Information (NCBI) Conserved Domain Database (CDD) [40].

Afterwards, *L*. *dendroidea* specimens obtained from an unialgal culture (see 38 for details) were frozen in liquid nitrogen and ground to a fine powder using a mortar and pestle. Total RNA was extracted with the Qiagen RNeasy^®^ Mini Kit (Hilden, Germany) and cDNA was prepared with the Bio-Rad iScript^TM^ cDNA Synthesis Kit (Hercules, United States). The full-length coding sequence of *LdCAS* was amplified from this *L*. *dendroidea* cDNA using the primers *LdCAS*_Fw and *LdCAS*_RV (Table 1). The obtained PCR fragment was Gateway^TM^ recombined into the Gateway^TM^ vector pDONR207 and the resulting entry clone was sequence verified and further recombined into the in-house generated destination vector pESC-URA-tHMG1-DEST [41] to yield pESC-URA-tHMG1-DEST[*GAL1/LdCAS*].

**Table 1:** Primer sequences used in this study.

Oligo	Sequence	Description
combi1715	5’-TAATACGACTCACTATAGGG-3’	T7 sequencing primer
combi2287	5’-GGAATAAGGGCGACACGG-3’	bla internal Rv
combi3244	5’-GTTAACCGGCCGCAAATTAAAGCC-3’	HpaI-CYC1t Rv
combi3245	5’-ggggacaagtttgtacaaaaaagcaggcttaAAGGGAACAAAAGCTGGAGC-3’	attB1-SNR52p Fw
combi3246	5’-ggggaccactttgtacaagaaagctgggtaAAAGCCTTCGAGCGTCCC-3’	attB2-CYC1t Rv
combi3247	5’-GTTAAC GCTAGCGAGGGAACAAAAGCTGGAGC-3’	HpaI-NheI-TEFp Fw
crispr014	5’-AGAGTTCCTCGGTTTGCCGATCATTTATCTTTCACTGCGGAGAAG-3’	TRP1 gRNA left Rv
crispr031	5’-GGCAAACCGAGGAACTCTGTTTTAGAGCTAGAAATAGCAAGTTAAAATAAGG-3’	TRP1 gRNA right fw
crispr059	5’-AACTGCATGGAGATGAGTCGTGGCATTAATAACAGAGTTCCTCGGTTTGCCAGTTATT-3’	TRP1 HR donor Fw
crispr060	5’-AATAACTGGCAAACCGAGGAACTCTGTTATTAATGCCACGACTCATCTCCATGCAGTT-3’	TRP1 HR donor Rv
crispr119	5’-AGGTAGTTCTGGTCCATTGG-3’	TRP1 RFLP fw
crispr120	5’-ACACCATTTGTCTCCACACC-3’	TRP1 RFLP rev
*LdCAS*_Fw	5’-GGGGACAAGTTTGTACAAAAAAGCAGGCTTAATGGTTGTTTGGCGACTCAATG-3’	*LdCAS* fw
*LdCAS*_RV	5’-GGGGACCACTTTGTACAAGAAAGCTGGGTATTATTGCTGCTGGCACGCTTTC-3’	*LdCAS* rev

### Generation and Culturing of *Saccharomyces cerevisiae* (Yeast) Strains

All primers used to create novel yeast strains and all yeast strains created and/or used in this study are listed in Table 1 and Table 2, respectively. Yeast strains PA14, TM097, and TM122 were derived from strain TM1 in the S288c BY4742 background (26).

**Table 2:** Yeast strains used in this study.

Strain	Genotype	Reference
*S288c BY4742*	MATα his3Δ1 leu2Δ0 lys2Δ0 ura3Δ0	Moses *et al*., 2014
TM1	S288c BY4742; P_*erg7*_::P_*MET3*_-*ERG7*	Moses *et al*., 2014
PA14	TM1; trp1Δ0	This study
TM097	PA14; pESC-URA-tHMG1-DEST	This study
TM122	PA14; pESC-URA-tHMG1-DEST[*GAL1*/*LdCAS*]	This study

Yeast strain PA14 was derived from strain TM1 [21] by knocking out the TRP1 gene using CRISPR/Cas9. A plasmid for CRISPR/Cas9 in yeast that contains both Cas9 and a gRNA cassette was generated according to the system reported by DiCarlo and co-workers [42]. To this end, the Cas9 expression cassette was PCR-amplified from p414-TEF1p-Cas9-CYC1t (Addgene plasmid 43802) using primers combi3244 and combi3247, each containing a *Hpa*I restriction site at the 5’ terminus. The resulting fragment was cloned into pJET1.2 and sequence verified. Subsequently, the Cas9 cassette was cut out using *Hpa*I, gel-purified and cloned into the vector backbone of a *Pvu*II-treated, dephosphorylated, and gel-purified pESC-URA plasmid (Agilent). The resulting plasmid was named pCAS1. Next, a Gateway^TM^ cassette was PCR-amplified from pDEST14 (Invitrogen, Carlsbad, United States) using primers combi1715 and combi2287. The PCR fragment was treated with *Xba*I and *Nhe*I, gel purified and cloned into the *Nhe*I-linearized and dephosphorylated pCAS1, yielding pCAS-ccdB. The TRP1 knock-out construct was generated by PCR amplification of SNR52p and sgRNA-CYC1t from p426-SNR52p-gRNA.CAN1.Y-SUP4t (Addgene plasmid 43803) using primers combi3245 and crispr014 and combi3246 and crispr031, respectively. The individual fragments were fused by overlap extension PCR, subcloned into pDONR221, sequence verified and finally Gateway^TM^ recombined into pCAS-ccdB, yielding pCAS-TRP1. Subsequently, 200 ng of pCAS-TRP1 and 10 μmol of double-stranded DNA (prepared by annealing the single-stranded oligonucleotides crispr059 and crispr060) as homologous recombination donor were co-transformed in yeast strain TM1. The resulting colonies were analyzed for positive CRISPR events by replica plating on SD medium (Clontech, Mountain View, United States) with or without tryptophan. Tryptophan auxotrophs were further confirmed by Sanger sequencing. Auxotrophic strains were cured of pCAS-TRP1 by counter-selection on plates containing 1 mg/mL 5-fluoroorotic acid (Zymo Research, Irvine, United States) and the resulting TM1-derived trp1 strain was named PA14.

Subsequently, the PA14 strain was transformed with the *LdCAS* expression construct or the empty pESC-URA-tHMG1-DEST vector to yield strain TM122 and the control strain TM097, respectively.

The yeast strains TM097 and TM122 were cultivated in the presence of methyl β-cyclodextrin (MβCD) as described [30]. On day 1, for each strain five individual yeast colonies were used to inoculate 5 mL of synthetic defined (SD) medium containing glucose with the–Ura dropout (DO) supplement (Clontech). The pre-cultures were grown for 24 h at 30°C with agitation (200 rpm). To induce heterologous gene expression, the pre-cultures were washed with 1 mL of sterile water and used to inoculate 15 mL of SD Gal/Raf medium containing galactose and raffinose with the–Ura DO supplement (Clontech). The induced cultures were incubated for 24 h and on day 3, methionine and MβCD were added to 1 mM and 5 mM, respectively. After a further 24 h incubation, MβCD was added once again to 5 mM. After a final 24 h incubation, the yeast cultures were stored at 4°C for three days and on day 8, 1 mL of each culture was extracted thrice with 500 μL of hexane. The pooled organic fractions were evaporated and derivatized with 10 μL of pyridine (Sigma-Aldrich, St. Louis, United States) and 50 μL of *N*-methyl-*N*-(trimethylsilyl)trifluoroacetamide (Sigma-Aldrich) prior to gas chromatography-mass spectrometry (GC-MS) analysis. The authentic cycloartenol standard (Sigma-Aldrich) was derivatized following the same method.

### Sterol extraction from *L*. *dendroidea*

Dried *L*. *dendroidea* material was ground to a fine powder under liquid nitrogen. Five milligram of the obtained powder was saponified by boiling it for 2 hours in 500 μL of 40% KOH and 500 μL of 50% EtOH. The resulting mixture was extracted thrice with 500 μL of hexane. The organic fractions were pooled, evaporated *in vacuo* and derivatized with 10 μL of pyridine (Sigma-Aldrich, St. Louis, United States) and 50 μL of *N*-methyl-*N*-(trimethylsilyl)trifluoroacetamide (Sigma-Aldrich, St. Louis, United States) prior to GC-MS analysis. An authentic cholesterol standard (Sigma-Aldrich, St. Louis, United States) was derivatized following the same method.

### GC-MS analysis

GC-MS analysis was performed using a GC model 6890 and MS model 5973 (Agilent, Santa Clara, United States). A VF-5ms capillary column (Varian CP9013, Agilent) was operated at a constant helium flow of 1 mL/min and 1 μL of the sample was injected in splitless mode. The oven was initially held at 80°C for 1 min, ramped to 280°C at a rate of 20°C/min, held at 280°C for 45 min, ramped to 320°C at a rate of 20°C/min, held at 320°C for 1 min, and at the end of the run cooled to 80°C at a rate of 50°C/min. Throughout the analysis, the injector was set to 280°C, the MS transfer line to 250°C, the MS ion source to 230°C, and the quadrupole to 150°C. A full electron ionization (EI) mass spectrum was generated by scanning the m/z range of 60–800 with a solvent delay of 7.8 min.

### Phylogenetic analysis

All OSC sequences reported in [26] were downloaded from the Phytozome database (*A*. *thaliana*) and NCBI (other species). Additional OSC sequences released after this study were screened in literature and included in our phylogenetic analysis. Subsequently, a Python script [43, 44] was used to obtain only the coding sequences to facilitate the alignment. The coding sequences were translated into amino acids and aligned using the software SeaView [45] employing the Clustal Omega algorithm [46]. After this, we constructed a neighbour joining tree using the Kimura 2 parameter model [47] with 1,000 bootstrap replicates. In the end, the tree was edited using FigTree.

## Results and Discussion

### Identification of *LdCAS*

Mining of *L*. *dendroidea* transcriptome data [38] revealed only one full-length OSC with an open reading frame of 739 amino acids. This OSC was cloned from *L*. *dendroidea* cDNA and named *LdCAS*. The *LdCAS* sequence was submitted to GenBank (accession number KX343073). The search for *LdCAS* conserved domains returned one hit, the squalene cyclase (SQCY) domain subgroup 1 (NCBI CDDS ID: cd02892), which has an alpha 6 –alpha 6 barrel fold and belongs to the Isopren C2 like superfamily (NCBI CDDS ID: cl08267). Further, using the *CAS1* from *A*. *thaliana* (UniProtKB ID: P38605), we were able to identify all five farnesyltransferase B subunit (PFTB) repeats in *LdCAS*. The mutagenesis sites, where the change of residues leads to the production of distinct compounds, and the active sites of *AtCAS1* and *LdCAS* were all the same, suggesting that our *LdCAS* is indeed a cycloartenol synthase, as *AtCAS1* is the cycloartenol synthase from *A*. *thaliana*, previously isolated and characterized [29].

**Fig 1:**
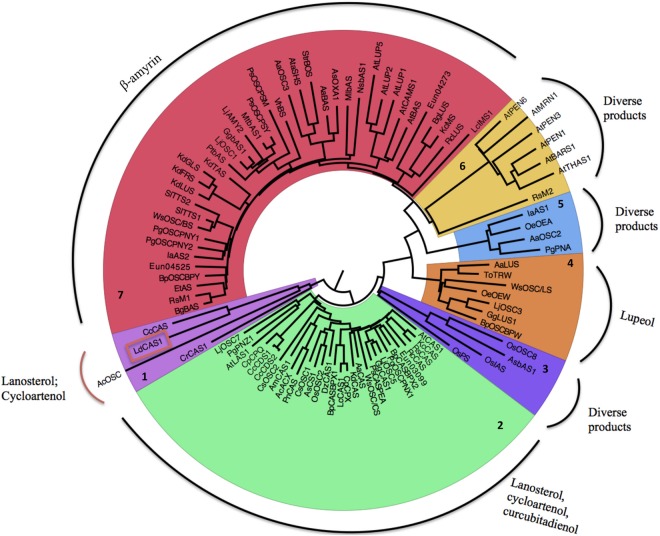
Neighbour-joining tree of OSCs from several organisms. OSC amino acid sequences (S1 Table) were aligned using Clustal Omega [59] with default parameters as implemented in the program SeaView [45] and all gaps were eliminated. The molecular evolutionary model chosen was Kimura 2 parameter [47]. The tree was reconstructed in SeaView with 1,000 bootstrap replicates. The name of each tip is the OSC name (source on S1 Table). Usually the first two characters represent the name of the species and the last three the product.

A phylogenetic tree using the amino acid sequences from putative OSCs from other species formed seven major groups (Fig 1). LdCAS clustered together with the OSCs from *Chondrus crispus*, a red seaweed, and with fungi and green algae OSCs (Fig 1, cluster 1). It is postulated that ergosterol is the final product of the CAS-dependent sterol biosynthesis pathway found in the genome of the green algae *Chlamydomonas reinhardtii*, as observed in fungi, despite the absence of the MVA pathway in green algae [48]. Since there is no other study that functionally characterized an OSC from red seaweeds, we could suggest that cycloartenol is the product of the OSC in red seaweeds.

The genes coding for cycloartenol, lanosterol and cucurbitadienol synthases were all grouped together (Fig 1, cluster 2). These products are generated from the same precursor–the protesterol cation. The modification of the protesterol cation by carbon removal leads to lanosterol and cycloartenol (see [49, 50] for further details). The modification of this molecule with a second methyl and hydrogen migration leads to curcubitadienol [51]. A third group of OSCs (Fig 1, cluster 7) consisted mainly of β-amyrin synthases. Other enzymes found within this group produce lupeol and mixed products. The phylogenetic tree showed four other minor groups. One group (Fig 1, cluster 4) was formed by some lupeol synthases and other enzymes, while another group (Fig 1, cluster 6) is composed mainly by *A*. *thaliana* enzymes. The last two groups (Fig 1, clusters 3 and 5) are mainly constituted by a broad group of multifunctional OSCs, which make a range of cyclization products.

The division of the OSC family according their product and higher rank phylogeny is similar to that observed in other studies. Xue *et al*. [28] divided the OSC family of plants into ten groups based on this pattern: pentacyclic triterpenes, lanosterol, cucurbitadienol, cycloartenol and unknown function within dicots and pentacyclic triterpene, isoarborinol, parkeol, cycloartenol and unknown function within monocots. Gas-Pascual *et al*. [52] included species from other kingdoms and found seven distinct OSC groups: lupeol, β-amyrin, promiscuous β-amyrin, plant lanosterol and cycloartenol from plants, protists and algae plus planctomycete. Together, these studies indicate that functional characterization is necessary to properly define the product specificity of an OSC since some phylogenetic groups have more than one product or because they are composed of multifunctional OSCs.

### Functional characterization of *LdCAS* in *S*. *cerevisiae*

To determine the enzymatic activity of *LdCAS*, we carried out functional characterization by means of heterologous expression in the yeast *S*. *cerevisiae*. Therefore, we first generated a *S*. *cerevisiae* strain capable of inducing terpene hyperproduction, as described previously [30, 53]. The yeast strain that we used was PA14, which is a modified version of the previously generated TM1 strain [30], containing an additional auxotrophic selection marker (see Material and Methods) and generated using the CRISPR/Cas9 genome editing tool [42]. As in the TM1 strain, the lanosterol synthase (ERG7) promoter in strain PA14 was replaced by a methionine repressible promoter. Addition of methionine to the yeast cultivation medium reduces the biosynthetic flux towards the sterols, leading to the accumulation of 2,3-oxidosqualene that can be used as a substrate for heterologously expressed OSCs. Additionally, a truncated feedback-insensitive version of the *S*. *cerevisiae* 3-hydroxy-3-methylglutaryl-CoA reductase 1 (tHMG1) enzyme was expressed from the high-copy-number plasmid pESC-URA to further increase the flux through the MVA pathway and thus further enhance the 2,3-oxidosqualene pool available for the heterologously expressed OSC.

**Fig 2:**
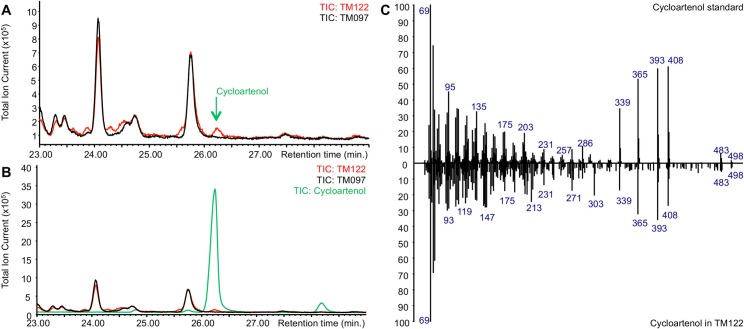
Expression of *LdCAS* in *S*. *cerevisiae* leads to the production of cycloartenol. (A) Overlay of GC-MS chromatograms from spent medium of the control yeast strain TM097 (black) and yeast strain TM122 expressing *LdCAS* (red). A peak unique to strain TM122 was observed with a retention time of 26.2 minutes. (B) Overlay of GC-MS chromatograms from spent medium of the control yeast strain TM097 (black) and yeast strain TM122 expressing *LdCAS* (red) and the GC-MS chromatogram of an authentic cycloartenol standard (green). The peak unique to strain TM122 has the same retention time as the authentic cycloartenol standard. (C) Comparison of the EI-MS spectra of the authentic cycloartenol standard (top) and the cycloartenol produced in strain TM122 (bottom).

Two yeast strains were generated from strain PA14. Strain TM122 expressed both tHMG1 and *LdCAS* from the pESC-URA-tHMG1-DEST plasmid, whereas the control strain TM097 only expressed tHMG1 from the pESC-URA-tHMG1-DEST plasmid. Both yeast strains were cultivated in the presence of MβCD as described [30]. After yeast cultivation, the extract of the spent medium of the TM122 strain was checked by GC-MS and compared to the extract of the spent medium of the control strain TM097. This analysis revealed the presence of a chromatographic peak unique to strain TM122 at 26.2 min with the same retention time and electron ionization (EI) spectrum as an authentic cycloartenol standard (Fig 2). The finding that *LdCAS* encodes a cycloartenol synthase is further underscored by previous studies suggesting cycloartenol to be the precursor of all sterols in marine seaweeds [54].

### Sterol profile of *L*. *dendroidea* and implications on sterol synthesis in red seaweeds

**Fig 3:**
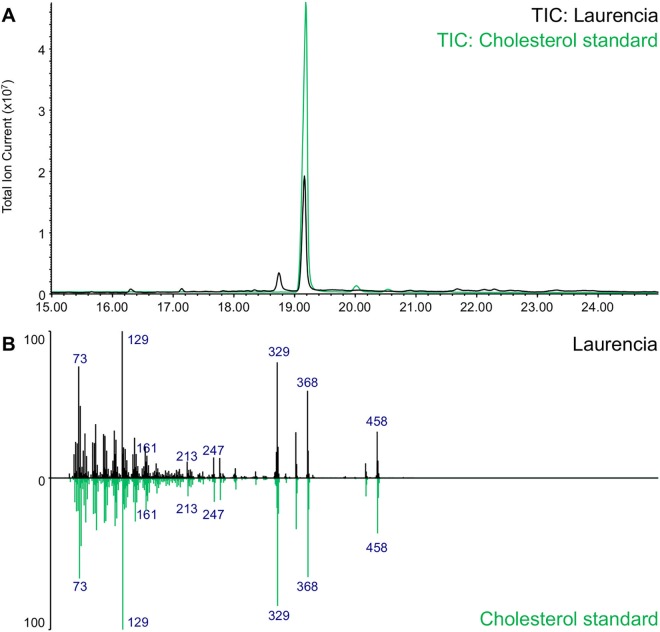
Sterol profiling of *Laurencia dendroidea* using GC-MS. (A) Total Ion Current chromatogram of the sterol extract of *L*. *dendroidea* (black) compared to an authentic cholesterol standard (green). (B) Comparison of the EI-MS spectra of cholesterol detected in *L*. *dendroidea* (black) and the authentic cholesterol standard (green).

As primary metabolites, sterols are essential structural components of cell membranes. Cholesterol, the main animal sterol, provides structural integrity and fluidity to the cell membrane. In fungi, the major sterol is erogsterol. Campesterol, stigmasterol and sitosterol are the most abundant phytosterols. Like for animals, the main sterol in red seaweeds was reported to be cholesterol [55]. To validate the sterol composition of the red seaweed *L*. *dendroidea*, sterol profiling was carried out by GC-MS (Fig 3). This analysis confirmed that, like other Rhodophyta, *L*. *dendroidea* accumulates cholesterol as its major sterol.

As the phytosterol precursor cycloartenol is the cyclization product of 2,3-oxidosqualene, and the major accumulating sterol in red seaweeds is the ‘animal’ sterol cholesterol, the sterol biosynthesis in red seaweeds may be a hybrid pathway between the plant and animal sterol synthesis pathways. Notably, unlike other plants, Solanaceae plant species also accumulate cholesterol as a major sterol [56, 57]. The biosynthesis of cholesterol in Solanaceae has not been elucidated yet, but was shown to involve a sterol side chain reductase enzyme (SSR) 2 that catalyzes the conversion of cycloartenol into cycloartanol by reduction of the C-24(25) double bond. This reduction step forms the branch point between the synthesis of phytosterols and cholesterol in Solanaceae [56] and it could thus be postulated that a similar reduction step takes place in the cholesterol synthesis of red seaweeds. Notably, this SSR2 enzyme seems to have arisen from DWF1 [56], a reductase enzyme involved in phytosterol synthesis that catalyzes the reduction of C-24(28) double bonds in C-24 alkylsterols and that is homologous to 24-dehydrocholesterol reductase (DHCR24), the animal C-24(25) reductase that catalyzes the conversion of desmosterol into cholesterol [58]. Hence, a key step in the synthesis of cholesterol in red algae may be the reduction of the C-24(25) double bond by a DHCR24-like enzyme.

## Conclusion

We were able to clone and characterize for the first time an OSC enzyme from a red seaweed. Through phylogenetic analysis we postulated that the product generated by *LdCAS* of *L*. *dendroidea* is cycloartenol. This finding was confirmed by the functional characterization of *LdCAS* in yeast and literature reports suggesting that cycloartenol is the sterol precursor of seaweed sterols. Furthermore, sterol profiling revealed that cholesterol is the major *L*. *dendroidea* sterol, implicating sterol synthesis in red seaweeds may be carried out via a hybrid of the plant and animal sterol synthesis pathways.

## Supporting Information

S1 Table OSCs IDs, species, reference number, product and source, if not in [1], of the data used in this work. (DOCX)
