# Supplementary material for: Cloning and Functional Characterization of Cycloartenol Synthase from the Red Seaweed Laurencia dendroidea
Source: PLoS One. 2016 Nov 10;11(11):e0165954. doi: 10.1371/journal.pone.0165954 (PMC5104453; doi:10.1371/journal.pone.0165954)
Supplement: S1 Table — (DOCX) [file pone.0165954.s001.docx]

**Supporting information**

**S1 Table.** OSCs IDs, species, reference number, product and source, if not in [1], of the data used in this work.

| **OSC ID** | **Species** | **GenBank** | **Product** | **Source** |
| --- | --- | --- | --- | --- |
| *PgOSCPNX1* | *Panax ginseng* | AB009029 | Cycloartenol |  |
| *PgOSCPNY1* | *Panax ginseng* | AB009030 | β-Amyrin |  |
| *PgPNZ1* | *Panax ginseng* | AB009031 | Lanosterol |  |
| *PgOSCPNY2* | *Panax ginseng* | AB014057 | β-Amyrin |  |
| *OeOEW* | *Olea europaea* | AB025343 | Lupeol |  |
| *ToTRW* | *Taraxacum officinale* | AB025345 | Lupeol |  |
| *GgCAS1* | *Glycyrrhiza glabra* | AB025968 | Cycloartenol |  |
| *LcCAS1* | *Luffa cylindrica* | AB033334 | Cycloartenol |  |
| *PsOSCPSY* | *Pisum sativum* | AB034802 | β-Amyrin |  |
| *PsOSCPSM* | *Pisum sativum* | AB034803 | Mixed_products | |
| *GgbAS1* | *Glycyrrhiza glabra* | AB037203 | β-Amyrin |  |
| *BpCASBPX1* | *Betula platyphylla* | AB055509 | Cycloartenol |  |
| *BpCASBPX2* | *Betula platyphylla* | AB055510 | Cycloartenol |  |
| *BpOSCBPW* | *Betula platyphylla* | AB055511 | Lupeol |  |
| *BpOSCBPY* | *Betula platyphylla* | AB055512 | β-Amyrin |  |
| *CcCAS* | *Chondrus crispus* | XM05715516 |  | [2] |
| *CsOSC1* | *Costus speciosus* | AB058507 | Cycloartenol |  |
| *CsOSC2* | *Costus speciosus* | AB058508 | Mixed_products | |
| *LcIMS1* | *Luffa cylindrica* | AB058643 | Isomultiflorenol | |
| *GgLUS1* | *Glycyrrhiza glabra* | AB116228 | Lupeol |  |
| *CpCPX* | *Cucurbita pepo* | AB116237 | Cycloartenol |  |
| *CpCPQ* | *Cucurbita pepo* | AB116238 | Cucurbitadienol | |
| *LjOSC1* | *Lotus japonicus* | AB181244 | β-Amyrin |  |
| *LjOSC3* | *Lotus japonicus* | AB181245 | Lupeol |  |
| *LjOSC5* | *Lotus japonicus* | AB181246 | Cycloartenol |  |
| *EtAS* | *Euphorbia tirucalli* | AB206469 | β-Amyrin |  |
| *LjOSC7* | *Lotus japonicus* | AB244671 | Lanosterol |  |
| *KcMS* | *Kandelia candel* | AB257507 | Mixed_products | |
| *RsM1* | *Rhizophora stylosa* | AB263203 | Mixed_products | |
| *RsM2* | *Rhizophora stylosa* | AB263204 | Mixed_products | |
| *PgPNA* | *Panax ginseng* | AB265170 | Dammarenediol | |
| *BgBAS* | *Bruguiera gymnorrhiza* | AB289585 | β-Amyrin |  |
| *BgLUS* | *Bruguiera gymnorrhiza* | AB289586 | Lupeol |  |
| *OeOEA* | *Olea europaea* | AB291240 | α-Amyrin |  |
| *RsCAS* | *Rhizophora stylosa* | AB292608 | Cycloartenol |  |
| *KcCAS* | *Kandelia candel* | AB292609 | Cycloartenol |  |
| *AcACX* | *Adiantum capillus-veneris* | AB368375 | Cycloartenol |  |
| *StrBOS* | *Stevia rebaudiana* | AB455264 | Baccharis_oxide | |
| *PnCAS* | *Polypodiodes niponica* | AB530328 | Cycloartenol |  |
| *AtaSHS* | *Aster tataricus* | AB609123 | Shiononea |  |
| *AmCAS1* | *Abies magnifica* | AF216755 | Cycloartenol |  |
| *LjAMY2* | *Lotus japonicus* | AF478455 | Mixed_products | |
| *AsbAS1* | *Avena strigosa* | AJ311789 | β-Amyrin |  |
| *AsCS1* | *Avena strigosa* | AJ311790 | Cycloartenol |  |
| *MtbAS1* | *Medicago truncatula* | AJ430607 | β-Amyrin |  |
| *OsPS* | *Oryza sativa* | AK066327 | Parkeol |  |
| *OsIAS* | *Oryza sativa* | AK067451 | Isoarborinol |  |
| *OsOSC8* | *Oryza sativa* | AK070534 | Mixed_products | |
| *OsOSC2* | *Oryza sativa* | AK121211 | Cycloartenol |  |
| *DzCAS1* | *Dioscorea zingiberensis* | AM697885 | Cycloartenol |  |
| *AtLUP5* | *Arabidopsis thaliana* | At1g66960 | Mixed_products | |
| *AtPEN6* | *Arabidopsis thaliana* | At1g78500 | Mixed_products | |
| *AtBAS* | *Arabidopsis thaliana* | At1g78950 | β-Amyrin |  |
| *AtCAMS1* | *Arabidopsis thaliana* | At1g78955 | Mixed_products | |
| *AtLUP2* | *Arabidopsis thaliana* | At1g78960 | Mixed_products | |
| *AtLUP1* | *Arabidopsis thaliana* | At1g78970 | Mixed_products | |
| *AtCAS1* | *Arabidopsis thaliana* | At2g07050 | Cycloartenol |  |
| *AtLAS1* | *Arabidopsis thaliana* | At3g45130 | Lanosterol |  |
| *AtPEN1* | *Arabidopsis thaliana* | At4g15340 | Mixed_products | |
| *AtBARS1* | *Arabidopsis thaliana* | At4g15370 | Mixed_products | |
| *AtPEN3* | *Arabidopsis thaliana* | At5g36150 | Mixed_products | |
| *AtMRN1* | *Arabidopsis thaliana* | At5g42600 | Marneral |  |
| *AtTHAS1* | *Arabidopsis thaliana* | At5g48010 | Thalianol |  |
| *AsOXA1* | *Aster sedifolius* | AY836006 | β-Amyrin |  |
| *PsCASPEA* | *Pisum sativum* | D89619 | Cycloartenol |  |
| *RcLUS* | *Ricinus communis* | DQ268869 | Lupeol |  |
| *RcCAS* | *Ricinus communis* | DQ268870 | Cycloartenol |  |
| *VhBS* | *Vaccaria hispanica* | DQ915167 | β-Amyrin |  |
| *PtbAS* | *Chlamydomonas reinhardtii* | EDP09612 | Cycloartenol |  |
| *AaBAS* | *Polygala tenuifolia* | EF107623 | β-Amyrin |  |
| *NsbAS1* | *Artemisia annua* | EU330197 | β-Amyrin |  |
| *WsOSC/CS* | *Nigella sativa* | FJ013228 | β-Amyrin |  |
| *KdTAS* | *Kalanchoe daigremontiana* | HM623868 | Taraxerolc |  |
| *KdGLS* | *Kalanchoe daigremontiana* | HM623869 | Glutinolc |  |
| *KdFRS* | *Kalanchoe daigremontiana* | HM623870 | Friedelinc |  |
| *KdLUS* | *Kalanchoe daigremontiana* | HM623871 | Lupeol |  |
| *KdCAS* | *Kalanchoe daigremontiana* | HM623872 | Cycloartenol |  |
| *SlTTS1* | *Solanum lycopersicum* | HQ266579 | β-Amyrin |  |
| *SlTTS2* | *Solanum lycopersicum* | HQ266580 | Mixed_products | |
| *WsOSC/LS* | *Withania somnifera* | JQ728552 | Lupeol | [3] |
| *WsOSC/BS* | *Withania somnifera* | JQ728553 | β-Amyrin | [3] |
| *AaOSC2* | *Artemisia annua* | KF309252 | Mixed_products | [4] |
| *MlbAS* | *Maesa lanceolata* | KF425519 | β-Amyrin | [4] |
| *AcOSC* | *Antrodia cinnamomea* | KJ094413 | Mixed_products | [5] |
| *Eun-04525* | *Eugenia uniflora* | KJ826401 | β-Amyrin | [6] |
| *Eun-03099* | *Eugenia uniflora* | KJ826402 | Cycloartenol | [6] |
| *Eun-04273* | *Eugenia uniflora* | KJ826403 | β-Amyrin | [6] |
| *IaAS1* | *Ilex asprella* | KM111167 | Mixed_products | [7] |
| *IaAS2* | *Ilex asprella* | KM111168 | Mixed_products | [7] |
| *AaCAS* | *Artemisia annua* | KM670093 | Mixed_products | [4] |
| *AaLUS* | *Artemisia annua* | KM670094 | Mixed_products | [4] |
| *AaOSC3* | *Artemisia annua* | KM670095 | Mixed_products | [4] |
| *CcCDS1* | *Citrullus colocynthis* | KM821404 | Lanosterol | [8] |
| *CcCDS2* | *Citrullus colocynthis* | KM821405 | Mixed_products | [8] |
| *LdCAS* | *Laurencia dendroidea* | KX343073 | Cycloartenol | This work |
| *CrCAS1* | *Chlamydomonas reinhardtii* | XM_001689822 | Cycloartenol | [9] |

**References**

1. Thimmappa R, Geisler K, Louveau T, O'Maille P, Osbourn A. Triterpene biosynthesis in plants. Annu Rev Plant Biol. 2014;65:225-57. doi: 10.1146/annurev-arplant-050312-120229. PubMed PMID: 24498976.

2. Collén J, Porcel B, Carré W, Ball S, Chaparro C, Tonon T, *et al*. Genome structure and metabolic features in the red seaweed *Chondrus crispus* shed light on evolution of the Archaeplastida. Proc Natl Acad Sci U S A. 2013;110(13):5247-52.

3. Dhar N, Rana S, Razdan S, Bhat WW, Hussain A, Dhar RS, *et al*. Cloning and functional characterization of three branch point oxidosqualene cyclases from *Withania* *somnifera* (L.) Dunal. J Biol Chem. 2014;289(24):17249-67. doi: 10.1074/jbc.M114.571919. PubMed PMID: 24770414; PubMed Central PMCID: PMC4059165.

4. Moses T, Pollier J, Shen Q, Soetaert S, Reed J, Erffelinck M-L, *et al*. OSC2 and CYP716A14v2 Catalyze the Biosynthesis of Triterpenoids for the Cuticle of Aerial Organs of *Artemisia annua*. Plant Cell. 2015;27(1):286-301. doi: 10.1105/tpc.114.134486.

5. Lin YL, Lee YR, Tsao NW, Wang SY, Shaw JF, Chu FH. Characterization of the 2,3-Oxidosqualene Cyclase Gene from *Antrodia cinnamomea* and Enhancement of Cytotoxic Triterpenoid Compound Production. J Nat Prod. 2015;78(7):1556-62. doi: 10.1021/acs.jnatprod.5b00020. PubMed PMID: 26125648.

6. Guzman F, Kulcheski FR, Turchetto-Zolet AC, Margis R. De novo assembly of *Eugenia uniflora* L. transcriptome and identification of genes from the terpenoid biosynthesis pathway. Plant Sci. 2014;229:238-46. doi: 10.1016/j.plantsci.2014.10.003. PubMed PMID: 25443850.

7. Zheng X, Luo X, Ye G, Chen Y, Ji X, Wen L, *et al*. Characterisation of two oxidosqualene cyclases responsible for triterpenoid biosynthesis in *Ilex asprella*. Int J Mol Sci. 2015;16(2):3564-78. doi: 10.3390/ijms16023564. PubMed PMID: 25664861; PubMed Central PMCID: PMC4346913.

8. Davidovich-Rikanati R, Shalev L, Baranes N, Meir A, Itkin M, Cohen S, *et al*. Recombinant yeast as a functional tool for understanding bitterness and cucurbitacin biosynthesis in watermelon (*Citrullus* spp.). Yeast. 2015;32(1):103-14. doi: 10.1002/yea.3049. PubMed PMID: 25308777.

9. Merchant SS, Prochnik SE, Vallon O, Harris EH, Karpowicz SJ, Witman GB, et al. The *Chlamydomonas* genome reveals the evolution of key animal and plant functions. Science. 2007;318(5848):245-50. doi: 10.1126/science.1143609. PubMed PMID: 17932292; PubMed Central PMCID: PMC2875087.
